# Evaluation of toxic and essential metal content in selected children’s, adult, and herbal toothpastes available in Türkiye: a cross-sectional analytical study

**DOI:** 10.1186/s12903-026-09080-1

**Published:** 2026-07-10

**Authors:** Sena Ayyıldız, Ayşenur Altuğ Yıldırım

**Affiliations:** 1 Department of Pediatric Dentistry, Faculty of Dentistry, Cankırı Karatekin University, Çankırı, Türkiye; 2 Department of Restorative Dentistry, Faculty of Dentistry, Cankırı Karatekin University, Çankırı, Türkiye

**Keywords:** Dentifrices, Trace Elements, Elemental analysis, Health risk assessment, Toxic Metals

## Abstract

**Background:**

Toothpastes are widely used oral care products and may contain trace and heavy metals originating from raw materials or manufacturing processes. Continuous monitoring of both toxic and essential metal content is therefore important to ensure consumer safety, particularly for products intended for children.

**Materials and methods:**

Fifteen commercially available toothpaste products marketed in Türkiye were purchased and classified as children’s, adult, or herbal formulations. Samples were digested using microwave-assisted nitric acid digestion and analyzed for aluminum (Al), chromium (Cr), iron (Fe), nickel (Ni), copper (Cu), zinc (Zn), arsenic (As), cadmium (Cd), mercury (Hg), and lead (Pb) using inductively coupled plasma mass spectrometry (ICP-MS). Metal concentrations were compared according to target group, formulation type, and country of manufacture using non-parametric statistical tests.

**Results:**

Mercury and arsenic were below the detection limits in all samples. Cadmium and lead were detected at concentrations within internationally recommended safety limits, while nickel, chromium, copper, zinc, aluminum, and iron were reported at varying concentrations without direct comparison to specific regulatory threshold values due to the lack of established limits for these metals in toothpaste products. No statistically significant differences were observed between children’s and adult toothpastes or between herbal and conventional formulations (*p* > 0.05). However, toothpastes manufactured in Türkiye exhibited significantly higher chromium concentrations compared to imported products (*p* = 0.028).

**Conclusion:**

The evaluated toothpaste products contained low levels of potentially toxic metals, with concentrations remaining within accepted safety limits. The findings suggest general compliance with international regulations; however, periodic surveillance remains important, particularly for products intended for children.

**Supplementary Information:**

The online version contains supplementary material available at https://doi.org/10.1186/s12903-026-09080-1.

## Introduction

Oral health is an integral component of general well-being, and routine toothbrushing with toothpaste remains the most accessible and effective method for maintaining daily oral hygiene. Toothpastes are widely used across all age groups and exist in numerous formulations, including conventional fluoride-containing products and herbal preparations marketed as “natural” or “safe.” While these products provide therapeutic and cosmetic benefits, increasing attention has been directed toward their safety profiles, particularly regarding the presence of trace elements and heavy metals [1]. Heavy metals characterized by high atomic weight, elevated density, and the capacity for bioaccumulation and environmental persistence are of particular concern due to their well-documented toxicological effects even at low exposure levels [2].

Since toothpastes are used daily and in direct contact with the oral mucosa, the possibility of ingestion especially in children who have limited swallowing reflex control further increases the potential health risks [3]. Consequently, the evaluation of heavy metal content in toothpaste formulations is essential for ensuring consumer safety.

In recent years, the increasing consumer preference for “chemical-free” and plant-derived oral care products has accelerated the market growth of herbal toothpastes [4]. Although herbal toothpastes are generally marketed as safe due to their plant-based composition, their heavy metal content has not been adequately investigated. Moreover, concerns have been raised regarding the potential risk of heavy metal contamination associated with the cultivation and extraction processes of herbal raw materials [5].

Studies have reported the presence of toxic heavy metals such as lead, cadmium, and chromium in a wide range of non-food consumer products used across different age groups. In children, detectable levels of heavy metals have been identified in personal care products, including baby powders, shampoos, sunscreens, and creams, with risk assessments suggesting potential exposure concerns during early life stages [6, 7]. In addition, investigations on children’s toys and objects intended for handling or mouthing behavior have demonstrated the release of toxic heavy metals, including lead, cadmium, and chromium, particularly from plastic- and paint-based materials, highlighting multiple non-dietary exposure pathways in childhood [8, 9]. In adults, studies investigating cosmetic and personal care products such as facial creams, lotions, foundations, lipsticks, sun protection products, and hair dyes have reported detectable concentrations of toxic heavy metals including lead, cadmium, chromium, nickel, and arsenic, with several studies also demonstrating potential health risks based on exposure and risk assessment approaches such as hazard quotient and lifetime cancer risk models [10, 11]. Within this broader context, toothpaste represents a unique exposure source due to its direct contact with the oral cavity and the potential for unintentional ingestion, particularly in children, thereby reinforcing the importance of evaluating its elemental safety profile [12].

Globally, toothpastes are generally classified as cosmetic products rather than pharmaceuticals, despite their widespread daily use. Although no universal international limits exist specifically for toothpastes, regulatory authorities such as the Food and Drug Administration (FDA), Health Canada (HC), European Union (EU) and the Turkish Medicines and Medical Devices Agency (TİTCK) have proposed permissible limits for heavy metals in cosmetics [13].Similarly, in Türkiye, the Ministry of Health through the Turkish Medicines and Medical Devices Agency (TİTCK) has also published specific threshold limits [14]. Previous studies have reported inconsistent compliance with these limits, underscoring the need for region-specific evaluations [15–17].

Inductively coupled plasma–mass spectrometry (ICP-MS) is a highly sensitive multi-element analytical technique that enables the simultaneous determination of trace and ultra-trace elements with low detection limits and a wide linear dynamic range [18]. Owing to its accuracy, precision, and suitability for complex matrices, ICP-MS is widely employed for the assessment of toxic and essential metals in consumer products [19, 20].

Therefore, the present study aimed to quantitatively assess and compare concentrations of selected toxic and essential metals (lead (Pb), cadmium (Cd), nickel (Ni), chromium (Cr), mercury (Hg), arsenic (As), aluminum (Al), iron (Fe), zinc (Zn), and copper (Cu)) in children’s, adult, and herbal toothpastes marketed in Türkiye using inductively coupled plasma–mass spectrometry (ICP-MS).

## Materials and methods

### Study design and toothpaste samples

A total of 15 commercially available toothpaste samples were purchased from retail markets in several provinces of Türkiye and classified as children’s, adult, or natural/herbal formulations (Table 1). Samples were coded as S1–S15 to ensure consistency throughout the manuscript.

**Table 1:** Characteristics of the toothpaste samples

Sample ID	Target group	Formulation	Country of Origin
S1	Children	Herbal/Natural	Imported
S2	Children	Conventional	Imported
S3	Children	Herbal/Natural	Manufactured in Turkey
S4	Children	Conventional	Imported
S5	Children	Conventional	Imported
S6	Children	Herbal/Natural	Manufactured in Turkey
S7	Children	Herbal/Natural	Imported
S8	Children	Herbal/Natural	Manufactured in Turkey
S9	Adult	Herbal/Natural	Manufactured in Turkey
S10	Adult	Herbal/Natural	Manufactured in Turkey
S11	Adult	Conventional	Manufactured in Turkey
S12	Adult	Conventional	Manufactured in Turkey
S13	Adult	Conventional	Imported
S14	Adult	Conventional	Manufactured in Turkey
S15	Adult	Conventional	Imported

### Sample preparation and microwave digestion procedure

Samples were analyzed without pre-treatment. A 1 g aliquot of each toothpaste was weighed in triplicate and digested in Teflon vessels with 8 mL of suprapure nitric acid (HNO₃) using a microwave digestion system (Milestone D5, USA). Digested samples were diluted to a final volume of 15 mL with deionized water. This digestion protocol was selected to ensure complete mineralization of the toothpaste matrix and to enable accurate multi-element analysis.

### Classification of analyzed elements

In this study, the analyzed elements were classified as essential or potentially toxic based on their biological roles in humans. Al, Cr, Fe, Ni, Cu, and Zn were considered essential or physiologically relevant trace elements due to their involvement in various metabolic and enzymatic processes, although excessive exposure may lead to adverse effects. In contrast, As, Pb, Cd, and Hg were classified as potentially toxic elements, as they have no known beneficial role in the human body and may cause toxic effects even at low exposure levels [21, 22] .This classification was used to facilitate interpretation of exposure relevance and risk assessment outcomes.

### ICP-MS analytical procedure

Concentrations of Al, Cr, Fe, Ni, Cu, Zn, As, Cd, Hg, and Pb were determined by inductively coupled plasma–mass spectrometry (ICP-MS). Multi-element calibration solutions were used for ICP-MS calibration over the range of 5.0 × 10⁻⁴ to 1.0 ppm depending on the analyzed element. Calibration performance was evaluated using correlation coefficients (R²), limits of detection (LOD), and limits of quantification (LOQ). LOQ values were calculated as 3.3 × LOD. Analytical performance characteristics are presented in Table 2. Since certified reference material specific to toothpaste was unavailable, TÜBİTAK UME CRM 1201 (trace elements in spring water) was used for instrumental quality control. ICP-MS measurements were performed without internal standard addition. To minimize cross-contamination, the sampler probe was rinsed sequentially with ultrapure water, 2% HNO₃, and ultrapure water between measurements. All analyses were performed in triplicate. Metals were grouped into essential and toxic categories to allow clearer interpretation of potential exposure risk and comparison with previous toxicological literature.

**Table 2:** Operating parameters of the ICP-MS instrument used for heavy metal analysis

Parameter	Value	Description
Radiofrequency power (RF power)	1550 W	Power applied to generate the plasma
Nebulizer gas flow	0.96 L/min	Argon gas flow used to aerosolize the sample
Plasma gas flow	0.88 L/min	Argon flow rate required to sustain the plasma
Nebulizer pressure	3.01 bar	Pressure applied to the nebulizer for aerosol production
Dwell time	0.01 ms	Measurement time spent on each isotope
Spray chamber temperature	3.7 °C	Temperature of the spray chamber ensuring aerosol stability

### Statistical analysis

Statistical analyses were performed using SPSS v24.0 (IBM Corp., Armonk, NY, USA) at a 95% confidence level.Metal concentrations (²⁷Al, ⁵²Cr, ⁵⁷Fe, ⁶⁰Ni, ⁶⁶Zn, ¹¹¹Cd, and ²⁰⁸Pb; KED) were treated as continuous variables, and mean values of triplicate measurements were used. Data were expressed as mean ± standard deviation, and relative standard deviation (RSD). Due to non-normal distribution and limited sample size, group comparisons (children vs. adult, domestic vs. imported, and natural/herbal vs. conventional) were performed using the Mann–Whitney U test. Hg and As were excluded from statistical analysis as they were below detection limits in all samples, while copper was excluded due to detection in only two samples. Concentrations below the detection limit (ND) were treated as non-detects and excluded from statistical analysis; however, their occurrence was reported descriptively. All statistical analyses were performed at a significance level of *p* < 0.05.

### Health risk assessment

A screening-level health risk assessment was performed for Pb and Cd exposure associated with toothpaste ingestion using an approach adapted from a recent study published in 2025 [12]. The assessment methodology was based on estimated daily dose (ADD) calculations considering metal concentration in toothpaste, toothpaste ingestion rate, and brushing frequency. Separate exposure scenarios were evaluated for children and adults. For children, a toothpaste ingestion rate of 0.30 g/brushing and a brushing frequency of two times per day were used, whereas for adults, an ingestion rate of 0.04 g/brushing and a brushing frequency of two times per day were applied [12]. The ADD values (µg/day) were calculated using the following equation:

$$\mathrm{ADD}=\mathrm{C}\times\mathrm{IR}\times\mathrm{F}$$

where *C* represents the metal concentration in toothpaste (ppm), *IR* is the toothpaste ingestion rate (g/brushing), and *F* is the brushing frequency (times/day) [12].

Hazard quotient (HQ) values were calculated for Pb and Cd by dividing the estimated daily dose by the corresponding health guidance values (HGVs). HGVs of 0.5 µg/day for Pb and 4.1 µg/day for Cd were adopted from the California Office of Environmental Health Hazard Assessment (OEHHA) maximum allowable dose levels (MADLs) [23] HQ values below 1 were considered indicative of no significant non-carcinogenic health risk [12].

Hg and As were not included in the health risk assessment because both elements were below the analytical detection limits in all analyzed samples.

## Results

### Analytical performance of ICP-MS method

The analytical performance of the ICP-MS method was evaluated using calibration linearity, limits of detection (LOD), and limits of quantification (LOQ) values for each analyzed element. The analytical performance of the ICP–MS method was evaluated using calibration linearity, limits of detection (LOD), and limits of quantification (LOQ) values for each analyzed element. The calibration linearity, LOD, and LOQ results are presented in Table 3.

**Table 3:** Calibration linearity, limits of detection (LOD), and limits of quantification (LOQ) for analyzed elements

Element	*R*²	LOD (ppm)	LOQ (ppm)
Al	0.9991	1.917 × 10⁻³	6.325 × 10⁻³
Cr	0.9998	1.922 × 10⁻⁴	6.340 × 10⁻⁴
Fe	0.9980	2.384 × 10⁻³	7.866 × 10⁻³
Ni	0.9991	1.577 × 10⁻⁴	5.200 × 10⁻⁴
Cu	0.9962	8.560 × 10⁻⁵	2.820 × 10⁻⁴
Zn	0.9999	9.285 × 10⁻⁴	3.064 × 10⁻³
As	0.9985	N.D.	N.D.
Cd	0.9996	8.350 × 10⁻⁵	2.760 × 10⁻⁴
Hg	0.9996	N.D.	N.D.
Pb	0.9952	3.356 × 10⁻⁴	1.107 × 10⁻³

LOQ values were estimated as 3.3 × LOD

LOD and LOQ values were not available for As and Hg in the instrument output

Both essential and toxic metals were quantified in the toothpaste samples (Tables 5 and 6). The interpretation of the results was performed in reference to maximum permissible concentrations established by international regulatory authorities, as summarized in Table 4 [14, 24–26].

**Table 4:** The maximum permissible concentrations of these metals in toothpaste are defined by various international guidelines

Heavy Metal	FDA [24]	EU [26]	Canada [25]	Turkey [14]
Lead (Pb)	20 ppm	0.5 ppm	1 ppm	1 ppm
Arsenic (As)	3 ppm	0.1 ppm	0.5 ppm	0.5 ppm
Mercury (Hg)	1 ppm	0.5 ppm	0.2 ppm	0.2 ppm
Cadmium (Cd)	-	-	0.1 ppm	0.1 ppm

Tables 5 and 6 present the concentrations of heavy metals and trace elements measured in 15 toothpaste samples (S1–S15) by ICP-MS. Results are expressed as mean ± standard deviation and relative standard deviation (RSD, %). Values below the detection limit are indicated as not detected (N.D.).

**Table 5:** Concentrations of essential metals in toothpaste samples determined by ICP–MS, expressed as mean ± SD and RSD values (ppm)

Sample	Al (Mean ± SD; RSD%) ppm	Cr (Mean ± SD; RSD%) ppm	Fe (Mean ± SD; RSD%) ppm	Ni (Mean ± SD; RSD%) ppm	Cu (Mean ± SD; RSD%) ppm	Zn (Mean ± SD; RSD%) ppm
S1	32.260 ± 0.569 (1.76%)	0.108 ± 0.003 (2.53%)	5.384 ± 0.055 (1.03%)	0.044 ± 0.003 (7.59%)	N.D.	0.095 ± 0.013 (13.79%)
S2	1.250 ± 0.040 (3.23%)	0.019 ± 0.001 (5.67%)	48.778 ± 0.380 (0.78%)	0.154 ± 0.016 (10.37%)	0.249 ± 0.008 (3.03%)	N.D.
S3	4.977 ± 0.146 (2.94%)	0.116 ± 0.008 (6.83%)	12.903 ± 0.085 (0.66%)	0.092 ± 0.016 (10.37%)	N.D.	0.026 ± 0.007 (27.87%)
S4	1.920 ± 0.139 (7.26%)	0.048 ± 0.002 (4.36%)	0.345 ± 0.035 (10.01%)	0.010 ± 0.001 (6.94%)	N.D.	N.D.
S5	46.700 ± 0.702 (1.50%)	0.174 ± 0.010 (5.63%)	3.379 ± 0.128 (3.77%)	0.042 ± 0.007 (15.43%)	N.D.	N.D.
S6	48.186 ± 0.882 (1.83%)	0.268 ± 0.013 (4.98%)	6.017 ± 0.422 (7.02%)	0.055 ± 0.006 (11.13%)	N.D.	557.549 ± 18.969 (3.40%)
S7	2.912 ± 0.067 (2.31%)	0.055 ± 0.0001 (0.0002%)	2.538 ± 0.101 (3.99%)	0.013 ± 0.002 (12.02%)	N.D.	0.058 ± 0.006 (11.11%)
S8	3.266 ± 0.142 (4.34%)	0.062 ± 0.002 (3.33%)	6.575 ± 0.215 (3.26%)	0.043 ± 0.002 (12.02%)	N.D.	N.D.
S9	4.425 ± 0.090 (2.04%)	0.110 ± 0.008 (7.70%)	0.964 ± 0.059 (6.14%)	0.023 ± 0.002 (7.44%)	N.D.	0.553 ± 0.022 (4.02%)
S10	31.545 ± 0.283 (0.90%)	0.352 ± 0.006 (1.76%)	3.759 ± 0.198 (5.26%)	0.137 ± 0.010 (7.58%)	N.D.	994.730 ± 22.049 (2.22%)
S11	28.446 ± 0.534 (1.88%)	0.077 ± 0.002 (2.26%)	4.276 ± 0.107 (2.51%)	0.037 ± 0.002 (5.33%)	N.D.	0.401 ± 0.038 (9.49%)
S12	26.967 ± 0.591 (2.19%)	0.079 ± 0.002 (2.70%)	3.979 ± 0.235 (5.91%)	0.035 ± 0.003 (8.90%)	0.161 ± 0.007 (4.26%)	0.078 ± 0.012 (15.84%)
S13	3.450 ± 0.101 (2.92%)	0.022 ± 0.004 (18.16%)	0.543 ± 0.235 (5.91%)	N.D.	N.D.	N.D.
S14	33.680 ± 1.206 (3.58%)	0.136 ± 0.005 (3.82%)	4.920 ± 0.110 (2.23%)	0.043 ± 0.003 (8.90%)	N.D.	0.227 ± 0.043 (18.87%)
S15	4.405 ± 0.048 (1.08%)	0.017 ± 0.002 (11.93%)	0.120 ± 0.038 (31.92%)	N.D.	N.D.	N.D.

Values are expressed as mean concentrations (ppm). N.D.: not detected

Values are presented as mean ± standard deviation (SD) of triplicate measurements

Relative standard deviation (RSD) values are given in parentheses

**Table 6:** Concentrations of toxic metals in toothpaste samples determined by ICP–MS expressed as mean ± SD and RSD values (ppm)

Sample	As	Cd (Mean ± SD; RSD%) ppm	Hg	Pb (Mean ± SD; RSD%) ppm
S1	N.D.	0.00096 ± 0.00042 (43.30%)	N.D.	0.00583 ± 0.00121 (20.71%)
S2	N.D.	0.00145 ± 0.00072 (50.00%)	N.D.	N.D.
S3	N.D.	0.00096 ± 0.00083 (86.60%)	N.D.	0.00682 ± 0.00071 (10.36%)
S4	N.D.	N.D.	N.D.	N.D.
S5	N.D.	N.D.	N.D.	0.01878 ± 0.00034 (1.83%)
S6	N.D.	0.00578 ± 0.00331 (57.28%)	N.D.	0.12421 ± 0.00470 (3.78%)
S7	N.D.	N.D.	N.D.	0.00738 ± 0.00209 (28.35%)
S8	N.D.	N.D.	N.D.	N.D.
S9	N.D.	N.D.	N.D.	N.D.
S10	N.D.	0.00193 ± 0.00151 (78.06%)	N.D.	0.05102 ± 0.00317 (6.21%)
S11	N.D.	N.D.	N.D.	0.02048 ± 0.00152 (7.40%)
S12	N.D.	N.D.	N.D.	0.01334 ± 0.00167 (12.54%)
S13	N.D.	N.D.	N.D.	N.D.
S14	N.D.	N.D.	N.D.	0.01835 ± 0.00054 (2.94%)
S15	N.D.	N.D.	N.D.	N.D.

Values are expressed as mean concentrations (ppm). N.D.: not detected

Values are presented as mean ± standard deviation (SD) of triplicate measurements

Relative standard deviation (RSD) values are given in parentheses

Non-parametric analyses showed no statistically significant differences in metal concentrations between children’s and adult toothpastes or between natural/herbal and conventional formulations (all *p* > 0.05).

**Fig. 1:**
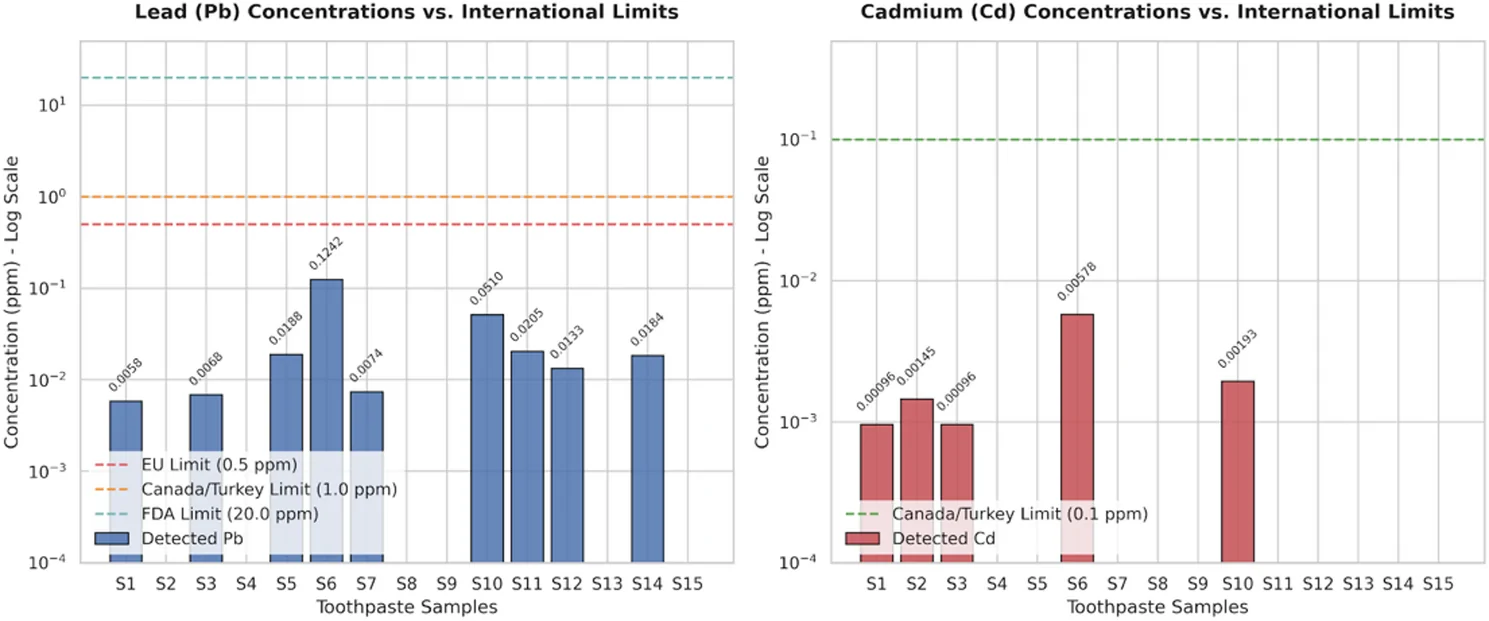
Comparison of detected heavy metal concentrations (Pb) and (Cd) in toothpaste samples against maximum permissible limits established by international regulatory agencies

Figure 1 presents the comparison of detected Pb and Cd concentrations with international regulatory guideline values. Arsenic (As) and mercury (Hg) were not included in the figure because their con centrations were below detectable levels in all analyzed samples.

Overall, Fe and Al were the most abundant elements across the analyzed toothpaste samples, whereas Cd, Pb, As, and Hg were either not detected or detected at very low frequencies. Ni, Cr, Zn, and Cu showed considerable inter-sample variability depending on formulation and product origin. These findings are consistent with the methodological detection limits and analytical performance characteristics of the ICP-MS method, confirming reliable quantification across all measurable elements. Statistical analysis indicated no significant differences in most elements between children’s and adult toothpastes or between natural/herbal and conventional formulations (*p* > 0.05). When stratified by country of manufacture, a significant difference was observed only for Cr (⁵²Cr, KED), with toothpastes produced in Türkiye exhibiting higher median Cr concentrations than imported products (*p* = 0.028). No significant differences were detected for the remaining elements. Detailed Mann–Whitney U test results are provided in Supplementary Tables S1–S3.

The screening-level health risk assessment demonstrated that the estimated daily dose (ADD) and hazard quotient (HQ) values for Pb and Cd were low in both children and adults across all analyzed toothpaste samples (Table 7). For Pb, the highest calculated HQ values were observed in sample S6, with values of 0.149 for children and 0.020 for adults, both remaining well below the threshold value of 1. Similarly, Cd exposure estimates and corresponding HQ values were extremely low in all samples, with the maximum child and adult HQ values calculated as 0.00085 and 0.00011, respectively. These findings indicate that the detected Pb and Cd concentrations in the analyzed toothpastes are unlikely to pose significant non-carcinogenic health risks under normal conditions of toothpaste use. Hg and As were not included in the risk assessment because their concentrations were below the detection limits in all samples.

**Table 7:** Screening-level health risk assessment of lead (Pb) and cadmium (Cd) exposure from incidental toothpaste ingestion in children and adults

Sample	Pb (ppm)	Pb Child ADD (µg/day)	Pb Child HQ	Pb Adult ADD (µg/day)	Pb Adult HQ	Cd (ppm)	Cd Child ADD (µg/day)	Cd Child HQ	Cd Adult ADD (µg/day)	Cd Adult HQ
S1	0.00583	0.00350	0.007	0.00047	0.0009	0.00096	0.00058	0.00014	0.00008	0.00002
S2	N.D.	N.C.	N.C.	N.C.	N.C.	0.00145	0.00087	0.00021	0.00012	0.00003
S3	0.00682	0.00409	0.008	0.00055	0.0011	0.00096	0.00058	0.00014	0.00008	0.00002
S4	N.D.	N.C.	N.C.	N.C.	N.C.	N.D.	N.C.	N.C.	N.C.	N.C.
S5	0.01878	0.01127	0.023	0.00150	0.0030	N.D.	N.C.	N.C.	N.C.	N.C.
S6	0.12421	0.07453	0.149	0.00994	0.0199	0.00578	0.00347	0.00085	0.00046	0.00011
S7	0.00738	0.00443	0.009	0.00059	0.0012	N.D.	N.C.	N.C.	N.C.	N.C.
S8	N.D.	N.C.	N.C.	N.C.	N.C.	N.D.	N.C.	N.C.	N.C.	N.C.
S9	N.D.	N.C.	N.C.	N.C.	N.C.	N.D.	N.C.	N.C.	N.C.	N.C.
S10	0.05102	0.03061	0.061	0.00408	0.0082	0.00193	0.00116	0.00028	0.00015	0.00004
S11	0.02048	0.01229	0.025	0.00164	0.0033	N.D.	N.C.	N.C.	N.C.	N.C.
S12	0.01334	0.00800	0.016	0.00107	0.0021	N.D.	N.C.	N.C.	N.C.	N.C.
S13	N.D.	N.C.	N.C.	N.C.	N.C.	N.D.	N.C.	N.C.	N.C.	N.C.
S14	0.01835	0.01101	0.022	0.00147	0.0029	N.D.	N.C.	N.C.	N.C.	N.C.
S15	N.D.	N.C.	N.C.	N.C.	N.C.	N.D.	N.C.	N.C.	N.C.	N.C.

*Abbreviations: ADD* average daily dose, *HQ* hazard quotient, *N.D.* not detected, *N.C.* not calculated

## Discussion

### Lead

Pb is a well-established toxic metal, and young children are considered particularly vulnerable due to their higher likelihood of toothpaste ingestion and increased neurodevelopmental sensitivity [27, 28]. Regulatory agencies therefore impose strict limits on lead in consumer products, with maximum allowable concentrations of 10 ppm recommended by the WHO and 1 ppm specified in the Turkish Cosmetics Regulation [29]. In the present study, lead was below the detection limit in 6 of the 15 toothpaste samples. In the remaining samples, Pb concentrations ranged from 5.83 to 124.21 ppb (0.00583–0.12421 ppm), which are well below international regulatory thresholds.

Compared with previously reported Pb levels in toothpastes, the concentrations observed in this study were substantially lower. For instance, Salama reported Pb levels ranging from1.856 to 6.313 ppm, while Vella and Attard in Malta found concentrations between 2.37 and 12.04 ppm. Studies in Saudi Arabia by Almukainzi et al. showed Pb levels of 8.8 to 9.19 ppm, and Lawi et al. reported a range of 1 to 12.05 ppm across ten samples. Furthermore, Paul et al. detected Pb concentrations of 0.27 to 2.12 ppm in Bangladesh, and Orisakwe reported levels as high as 23.575 mg/kg in Pepsodent and 18.092 mg/kg in Flodent [13, 15, 17, 30–32].

In a recent AAS-based study evaluating commercially available toothpastes, Pb concentrations were reported to range from below the limit of detection to 0.054 ppm, depending on formulation type, with most samples showing compliance with regulatory limits [33]. Similarly, a 2024 comparative analysis using LIBS and AAS techniques reported Pb concentrations of 0.34 ppm (LIBS) and 0.30 ppm (AAS) in one sample, and 0.524 ppm (LIBS) and 0.454 ppm (AAS) in another sample, indicating variability depending on analytical method and sample type [34].

In contrast, the maximum Pb concentration detected in the present study (0.124 ppm) was considerably lower than those reported in the global literature, indicating relatively low levels of lead contamination in toothpaste products marketed in Türkiye. Notably, only one sample showed a value slightly higher than the upper range reported in a recent study from India [33]. while all remaining samples were well below previously published concentrations.

Although median Pb concentrations were numerically higher in children’s toothpastes than in adult formulations, this difference was not statistically significant (*p* > 0.05), suggesting that lead presence is not strongly associated with product type. The presence of Pb in toothpaste products is generally attributed to trace contamination originating from raw materials and manufacturing processes, including mineral-based ingredients and environmental exposure during production [13, 35]. This explanation may account for the relatively low and non–formulation-specific distribution observed in the present study. Taken together, the findings indicate low levels of concern regarding lead exposure from toothpaste products currently available in Türkiye.

### Mercury and arsenic

Hg and As are well-recognized toxic elements, and their presence in cosmetic products is strictly regulated due to their potential adverse health effects, particularly in children [36, 37]. In the present study, both Hg and As were below the detection limits in all toothpaste samples analyzed. As no measurable variability was observed, these elements were excluded from statistical comparisons.

Previous studies have reported variable findings regarding Hg and As contamination in toothpaste products. Vella et al. similarly reported no detectable Hg levels in any of the nine toothpaste samples analyzed [13]. In contrast, Paul et al. reported As concentrations ranging from 0.02 to 0.637 ppm in toothpaste samples from Bangladesh, while Salama et al. detected As concentrations ranging from 0.60 to 26.94 ppm in four toothpaste samples [15, 17]. An AAS-based analysis of commercially available toothpastes from India also reported As concentrations ranging from below the detection limit to 0.54 ppm, with several products exceeding EU regulatory limits [1]. In addition, a recent comparative study using LIBS and AAS techniques detected Hg concentrations ranging from 0.319 to 0.320 ppm by LIBS and from 0.355 to 0.390 ppm by AAS in toothpaste samples [34].

The presence of Hg and As in cosmetic products has been associated with environmental contamination or their intentional or unintentional inclusion as impurities in raw materials used during manufacturing processes [35]. The consistent absence of Hg and As across all samples indicates good compliance with international regulatory standards and suggests that commercially available toothpastes in Türkiye pose minimal health risks related to Hg and As exposure.

### Cadmium

Cd is a toxic heavy metal, and chronic exposure has been associated primarily with renal toxicity [38, 39]. Its presence in consumer products is therefore strictly regulated [14, 24, 25, 40, 41]. Children are particularly vulnerable to the neurotoxic effects of Cd due to ongoing brain development, with studies linking Cd exposure to cognitive deficits and learning difficulties in this population [42, 43].

In the present study, Cd was below the detection limit in 10 of the 15 toothpaste samples, while the remaining samples showed Cd concentrations ranging from 0.00096 to 0.00578 ppm. These findings indicate minimal cadmium contamination in the analyzed toothpaste products. Similar observations have been reported in previous studies. Vella et al. detected no measurable Cd in any of the nine toothpaste samples evaluated, suggesting concentrations below the analytical detection threshold. Likewise, Orisakwe et al., in an analysis of 35 toothpaste products, reported Cd concentrations below the detection limit (< 0.001 mg/kg) in 9 samples. A recent comparative study using LIBS and AAS techniques also reported relatively low Cd concentrations ranging from 0.0101 to 0.037 ppm by LIBS and from 0.015 to 0.033 ppm by AAS in toothpaste samples. In contrast, higher Cd levels have been reported in some regions; Odukudu et al. reported Cd concentrations ranging from 0.010 to 0.070 ppm in Nigerian toothpaste samples, whereas Omosigho et al. reported Cd levels ranging from 2.470 to 2.952 ppm [13, 31, 34, 44, 45]. No statistically significant differences in Cd concentrations were observed between children’s and adult toothpastes, between natural/herbal and conventional formulations, or between domestically manufactured and imported products (*p* > 0.05). This is likely attributable to trace contamination from raw materials and environmental sources during production, including plant-derived ingredients, mineral-based components, and manufacturing-related contamination as previously reported in cosmetic products [35]. Overall, Cd contamination in toothpastes marketed in Türkiye was minimal and within internationally accepted safety limits [14, 24, 25, 40, 41].

### Nickel

Ni is a naturally occurring trace element and is generally regarded as an unavoidable contaminant in cosmetic products due to its presence in raw materials [24, 26].

In the present study, Ni was detected in 13 of the 15 toothpaste samples, with concentrations reaching a maximum of 0.154 ppm (153.78 ppb). Similar nickel levels have been reported in some previous studies. Valentine et al. reported Ni concentrations ranging from 0.00 ± 0.00 to 0.08 ± 0.02 ppm in toothpaste samples, while Odukudu et al. reported Ni concentrations ranging from 0.010 to 0.050 ppm.In contrast, higher Ni concentrations have also been documented. Vella et al. reported Ni levels ranging from 0.43 ppm in herbal toothpastes to 2.54 ppm in children’s toothpastes. Similarly, Salama et al. reported Ni concentrations ranging from 0.074 to 1.557 ppm in four different toothpaste samples [13, 16, 17, 44]. Compared with these findings, the Ni concentrations detected in the present study were generally lower than those reported in the literature.

The presence of Ni in toothpaste is mainly attributed to its occurrence as a trace impurity in mineral-based raw materials, as well as potential contamination introduced during manufacturing processes and water or equipment-related sources [1, 13].

No statistically significant differences in Ni concentrations were observed between children’s and adult toothpastes, between natural/herbal and conventional formulations, or between domestically manufactured and imported products (*p* > 0.05). The relatively uniform distribution of Ni across samples suggests background contamination rather than formulation-specific sources. Overall, the low Ni concentrations detected are unlikely to pose significant health risks.

### Chromium

Cr occurs primarily in two oxidation states: trivalent chromium [Cr(III)], which is considered an essential trace element, and hexavalent chromium [Cr(VI)], which is recognized as toxic and carcinogenic [38].

In the present study, Cr concentrations ranged from 0.01698 to 0.35245 ppm, which are lower than or comparable to levels reported in previous studies evaluating toothpaste products. Valentine et al. reported Cr concentrations ranging from 0.00 ± 0.00 to 1.89 ± 0.50 ppm in toothpaste samples, Odukudu et al. detected Cr concentrations ranging from 0.010 to 0.030 ppm. Higher Cr concentrations have also been documented in the literature. Vella et al. reported Cr levels ranging from 0.28 ppm in children’s toothpastes to 7.35 ppm in conventional formulations. Similarly, Salama et al. detected Cr concentrations ranging from 0.839 to 4.763 ppm in four different toothpaste samples. In addition, Almukainzi et al., in an analysis of cosmetic products including two toothpaste samples, reported Cr concentrations of 0.878 and 1.611 ppm [13, 16, 17, 30, 44]. Compared with these studies, the chromium concentrations observed in the present study were generally lower than those previously reported in the literature.

Statistical analysis revealed a significant difference in Cr concentrations according to the country of manufacture. Toothpastes produced in Türkiye exhibited significantly higher median ⁵²Cr (KED) levels than imported products (*p* = 0.028). In contrast, no statistically significant differences were observed between children’s and adult toothpastes or between natural/herbal and conventional formulations (*p* > 0.05).

Cr concentrations were significantly higher in Turkish-origin toothpaste samples compared with imported products. One possible explanation may involve differences in raw material sourcing and geological background characteristics. Türkiye contains extensive chromite deposits and widespread ophiolitic geological formations enriched with Cr -bearing ultramafic rocks [46, 47]. In addition, environmental studies from Türkiye have demonstrated regional variations in trace element composition of soils and plant materials associated with geological background characteristics [48]. These findings suggest that geographical and geological factors may influence the trace element profile of plant- or mineral-derived raw materials used in consumer products.

In the present study, several toothpaste samples with the highest Cr concentrations were marketed as herbal, natural, or fluoride-free formulations and contained multiple plant-derived oils, extracts, and mineral-based ingredients. Cr in cosmetic and personal care products is generally regarded as an unavoidable trace impurity that may originate from mineral ingredients, plant-derived materials, or manufacturing-related contamination during sourcing and production processes. Therefore, the relatively higher Cr concentrations observed in some Turkish-origin products may be influenced by variability in raw material sourcing and regional geological background characteristics, although further studies are needed to confirm this hypothesis.

### Copper

Cu is an essential trace element involved in numerous enzymatic and metabolic processes; however, excessive exposure may result in toxic effects [49, 50].

In the present study, Cu was detected in only 2 of the 15 toothpaste samples, with concentrations ranging from 0.161 to 0.249 ppm, while the remaining samples showed levels below the detection limit. Due to the limited number of detectable values, copper was excluded from statistical group comparisons. The presence of Cu in toothpaste formulations may be associated with its occurrence as a naturally present trace element in plant- and mineral-based raw materials, as well as its potential introduction through manufacturing processes and environmental contamination pathways [1, 13].

The Cu concentrations observed in this study were generally lower than those reported in previous investigations evaluating toothpaste products. Valentine et al. reported Cu concentrations ranging from 0.00 ± 0.00 to 1.83 ± 0.04 ppm in toothpaste samples, while Odukudu et al. detected Cu concentrations between 0.100 and 0.310 ppm. Vella et al. reported Cu levels ranging from 0.73 ppm in children’s toothpastes to 3.68 ppm in conventional formulations. Similarly, Salama et al. detected Cu concentrations ranging from 0.018 to 2.609 ppm in four different toothpaste samples. Compared with these findings, the Cu concentrations detected in the present study were relatively low, indicating limited copper contamination in the analyzed toothpaste products [13, 16, 17, 44]. Overall, the findings suggest that Cu exposure from toothpaste use in Türkiye is limited, although variability among products highlights the importance of continued monitoring.

### Iron

Fe is an essential trace element involved in numerous biological processes; however, excessive exposure may contribute to oxidative stress [38, 51].

In the present study, Fe was detected in all toothpaste samples, with concentrations ranging from 120.26 ppb (≈ 0.12 ppm) to 48777.67 ppb (≈ 48.78 ppm). Previous studies have also reported substantial variability in iron concentrations among toothpaste products. Vella et al. reported Fe concentrations ranging from 1.76 to 17.68 ppm, with both the minimum and maximum values observed in conventional toothpastes. Similarly, Valentine et al. detected Fe concentrations ranging from 0.00 ± 0.00 to 7.06 ± 0.12 ppm in toothpaste samples [13, 16]. Compared with these studies, the upper Fe concentrations observed in the present study were relatively higher, indicating variability in Fe content among commercially available toothpaste products.

Although median Fe concentrations were numerically higher in adult toothpastes and in products manufactured outside Türkiye, these differences were not statistically significant (*p* > 0.05). Although Fe is not typically included as a primary functional ingredient in toothpaste formulations, its presence may be associated with naturally occurring mineral impurities, abrasive components, pigment-containing ingredients, or trace contamination introduced during manufacturing and raw material processing [1, 13]. As no specific regulatory limit has been established for Fe content in toothpaste, the observed concentrations are best interpreted in the context of overall exposure, bioavailability, and frequency of use.

### Zinc

Zn is an essential element with important biological functions and is generally considered to have low toxicity under normal exposure conditions [52, 53]. Zn compounds, particularly zinc citrate, are frequently used in toothpaste formulations because of their antiplaque and antimicrobial properties [54].

In the present study, Zn was detected in 9 of the 15 toothpaste samples, with concentrations ranging from 26.44 ppb (≈ 0.026 ppm) to 994,729.76 ppb (≈ 994.73 ppm), indicating substantial inter-product variability. Previous studies have also demonstrated considerable variation in Zn concentrations across commercially available toothpaste products. Omosigho et al. reported Zn concentrations ranging from 13.396 to 24.164 ppm, while Valentine et al. found zinc levels between 0.00 ± 0.00 and 3.92 ± 0.89 ppm in toothpaste samples. Similarly, Odukudu et al. reported Zn concentrations ranging from 0.110 to 0.410 ppm [16, 44, 45]. Compared with these findings, the Zn concentrations observed in the present study show a much wider range, with some samples exhibiting substantially higher levels, indicating marked heterogeneity in Zn content among toothpaste formulations.

No statistically significant differences in Zn concentrations were observed between children’s and adult toothpastes, between natural/herbal and conventional formulations, or between domestically manufactured and imported products (*p* > 0.05). The observed variability likely reflects differences in formulation composition and the selective use of Zn-containing ingredients among products. Notably, sample S10, which exhibited the highest Zn concentration, listed zinc oxide among its ingredients, suggesting that the elevated zinc level was associated with the intentional use of a Zn -containing active ingredient rather than incidental contamination.

### Aluminum

In the present study, Al was detected in all toothpaste samples, with concentrations ranging from 1249.82 ppb (≈ 1.25 ppm) to 48186.01 ppb (≈ 48.19 ppm), indicating its widespread presence across different formulations. Comparable findings have been reported previously, and no FDA or USP limits have currently been established for Al in oral care products [30].

No statistically significant differences in Al concentrations were observed between children’s and adult toothpastes, between natural/herbal and conventional formulations, or between domestically manufactured and imported products (*p* > 0.05). The observed variability is therefore more likely related to formulation practices and raw material composition than to product category or labeling. Al compounds are commonly incorporated into toothpastes as mild abrasive agents or formulation components that contribute to cleaning efficacy and texture [55]. Overall, although Al was consistently detected, its uniform distribution across product groups underscores the importance of continued monitoring and the need for clearer regulatory guidance regarding Al content in toothpaste formulations.

### Health risk assessment

The screening-level health risk assessment performed in the present study demonstrated that the estimated daily exposures and hazard quotient (HQ) values for both Pb and Cd remained below the accepted safety thresholds in all evaluated samples for both children and adults. These findings are in agreement with a recent 2025 study reporting that although detectable concentrations of Pb, Cd, Hg, and As were identified in several toothpaste products, the calculated exposure doses generally remained below levels expected to cause significant toxicological risk when the products were used as directed [12]. In the current study, the low HQ values obtained for Pb and Cd further support the conclusion that the analyzed toothpaste products are unlikely to pose immediate non-carcinogenic health risks through routine use. Nevertheless, previous studies have demonstrated that toothpaste ingestion is more common in younger children and decreases with age, emphasizing that chronic low-level exposure through daily oral care products should still be monitored carefully, particularly in pediatric populations [3, 56].

## Conclusion

This study evaluated the heavy metal content of commercially available toothpastes in Türkiye, including products for children and adults as well as natural/herbal and conventional formulations. Overall, Cd, Pb, Ni, Cr, Cu, Zn, and Fe were detected at low concentrations, while Hg and As were below the detection limits in all samples. Where applicable, comparisons with internationally accepted safety limits indicated no exceedance for Cd, Pb, Hg, and As.

Statistical analyses showed no significant differences in metal concentrations according to age group or formulation type (natural/herbal vs. conventional), except for Cr, which varied significantly according to country of manufacture (manufactured in Turkey vs. imported), suggesting differences in raw material sources and/or manufacturing processes.

Although the detected levels are unlikely to pose immediate health risks, the cumulative exposure associated with daily toothpaste use, particularly among children, highlights the importance of continued monitoring. In this context, routine surveillance of heavy metal contents in toothpaste products and periodic regulatory screening are recommended to ensure ongoing consumer safety and compliance with international standards. The findings of this study may provide useful information for regulatory authorities and manufacturers in terms of monitoring heavy metal contamination in toothpaste products and ensuring compliance with cosmetic safety regulations.

In conclusion, the findings of this screening-level study suggest that toothpastes available on the Turkish market do not pose an immediate safety concern; however, larger-scale studies and continuous monitoring are warranted to ensure long-term consumer safety and regulatory compliance.

### Limitations

This study has certain limitations. The relatively small number of samples within each subgroup may have limited the statistical power to detect subtle differences in metal concentrations between groups (children vs. adults, herbal vs. conventional, and domestic vs. imported); therefore, the absence of statistically significant differences should be interpreted with caution, particularly because the risk of Type II error may be increased in pilot-scale studies with limited subgroup sizes. Additionally, batch-to-batch variability within the same toothpaste brands was not evaluated, and potential variations related to different production lots could not be assessed. Since heavy metal contamination may originate from raw material variability or manufacturing-related factors, analysis of a single sample per brand reflects only a snapshot of the product at the time of purchase and may not fully represent long-term manufacturing consistency. Future studies including larger sample sizes and multiple batches per product are warranted to provide a more comprehensive evaluation.

### Declaration of generative AI and AI-assisted technologies in the manuscript preparation process

During the preparation of this manuscript, the authors used ChatGPT (OpenAI) to assist with language editing, rephrasing of text, and improvement of clarity in selected sections. After using this tool, the authors critically reviewed, edited, and validated the content. The authors take full responsibility for the scientific content, data interpretation, and conclusions presented in this article.

## Supplementary Information

Supplementary Material 1.

## Data Availability

The datasets generated and/or analyzed during the current study are not publicly available due to institutional restrictions but are available from the corresponding author on reasonable request.
