# Supplementary material for: Evaluation of toxic and essential metal content in selected children’s, adult, and herbal toothpastes available in Türkiye: a cross-sectional analytical study
Source: BMC Oral Health. 2026 Jul 10;26:1912. doi: 10.1186/s12903-026-09080-1 (PMC13637093; doi:10.1186/s12903-026-09080-1)
Supplement: Supplementary file 1 [file 12903_2026_9080_MOESM1_ESM.docx]

**Supplemantary Tables**

**Table 1. Comparison of Metal Concentrations Between Adult and Children’s Toothpastes Using the Mann–Whitney U Test**

|  | Toothpaste | | | | | | | | | | Mann Whitney Test | |
| --- | --- | --- | --- | --- | --- | --- | --- | --- | --- | --- | --- | --- |
|  | Adult | | | | | Kids | | | | |  |  |
|  | X | ss | M | IQR | | X | ss | M | IQR | | U | p |
| 27Al (KED) | 19.743,63 | 21.807,05 | 4.977,14 | 1.920,41 | 46.699,60 | 17.022,98 | 14.187,95 | 15.696,14 | 3.927,58 | 29.995,30 | 27,000 | 0,908 |
| 52Cr (KED) | 112,66 | 85,73 | 107,76 | 48,44 | 173,72 | 106,80 | 107,04 | 77,95 | 41,71 | 122,85 | 26,000 | 0,817 |
| 57Fe (KED) | 11.334,84 | 16.978,29 | 5.383,73 | 2.538,29 | 12.903,20 | 3.141,96 | 2.328,19 | 3.868,85 | 753,43 | 4.597,94 | 19,000 | 0,298 |
| 60Ni (KED) | 58,60 | 50,15 | 43,70 | 13,43 | 92,42 | 52,85 | 41,90 | 39,84 | 34,83 | 42,91 | 18,000 | 0,668 |
| 66Zn (KED) | 139.432,16 | 278.744,63 | 76,55 | 42,31 | 278.822,01 | 199.197,81 | 444.715,91 | 400,90 | 227,41 | 553,27 | 5,000 | 0,221 |
| 111Cd (KED) | 2,29 | 2,34 | 1,21 | 0,96 | 3,62 | 1,93 |  | 1,93 | 1,93 | 1,93 | 1,000 | 0,468 |
| 208Pb (KED) | 32,60 | 51,48 | 7,38 | 6,82 | 18,78 | 25,80 | 17,08 | 19,42 | 15,85 | 35,75 | 6,000 | 0,327 |

**Table 2:Mann–Whitney U Test Results for the Comparison of Metal Concentrations in Toothpastes by Country of Origin**

|  | Country of Origin | | | | | | | | | | Mann Whitney Test | |
| --- | --- | --- | --- | --- | --- | --- | --- | --- | --- | --- | --- | --- |
|  | Turkish | | | | | Others | | | | |  |  |
|  | X | ss | M | IQR | | X | ss | M | IQR | | U | p |
| 27Al (KED) | 22.686,48 | 16.584,36 | 27.706,32 | 4.701,26 | 32.612,36 | 13.271,06 | 18.410,86 | 3.450,36 | 1.920,41 | 32.260,10 | 15,000 | 0,132 |
| 52Cr (KED) | 150,02 | 104,24 | 113,17 | 77,95 | 201,94 | 63,26 | 58,11 | 48,44 | 19,35 | 107,76 | 9,000 | 0,028 |
| 57Fe (KED) | 5.424,14 | 3.462,89 | 4.597,94 | 3.868,85 | 6.296,22 | 8.726,64 | 17.764,44 | 2.538,29 | 344,87 | 5.383,73 | 15,000 | 0,132 |
| 60Ni (KED) | 58,01 | 38,05 | 42,80 | 35,91 | 73,47 | 52,65 | 58,64 | 42,11 | 13,43 | 43,70 | 16,000 | 0,558 |
| 66Zn (KED) | 221.937,80 | 399.072,70 | 400,90 | 77,73 | 557.549,10 | 76,55 | 25,99 | 76,55 | 58,17 | 94,92 | 3,000 | 0,242 |
| 111Cd (KED) | 2,89 | 2,55 | 1,93 | 0,96 | 5,78 | 1,21 | 0,35 | 1,21 | 0,96 | 1,45 | 1,500 | 0,374 |
| 208Pb (KED) | 39,04 | 44,43 | 19,42 | 13,34 | 51,02 | 10,66 | 7,07 | 7,38 | 5,83 | 18,78 | 4,000 | 0,197 |

**Table 3. Mann–Whitney U test results for the comparison of metal concentrations in natural and conventional toothpaste formulations**

|  | Tootpaste classification | | | | | | | | | | Mann Whitney Test | |
| --- | --- | --- | --- | --- | --- | --- | --- | --- | --- | --- | --- | --- |
|  | Natural | | | | | Conventional | | | | |  |  |
|  | X | ss | M | IQR | | X | ss | M | IQR | | U | p |
| 27Al (KED) | 18.224,54 | 18.691,58 | 4.977,14 | 3.265,99 | 32.260,10 | 18.352,18 | 17.702,68 | 15.685,85 | 2.685,39 | 31.062,79 | 24,000 | 0,643 |
| 52Cr (KED) | 153,01 | 112,73 | 109,89 | 61,62 | 268,07 | 71,50 | 57,77 | 62,65 | 20,57 | 107,43 | 14,000 | 0,105 |
| 57Fe (KED) | 5.448,67 | 3.842,84 | 5.383,73 | 2.538,29 | 6.575,01 | 8.292,36 | 16.472,13 | 3.678,63 | 443,91 | 4.597,94 | 18,000 | 0,247 |
| 60Ni (KED) | 58,10 | 43,04 | 43,70 | 22,65 | 92,42 | 53,44 | 50,59 | 39,55 | 34,83 | 42,68 | 15,000 | 0,391 |
| 66Zn (KED) | 258.835,28 | 423.880,74 | 324,10 | 58,17 | 557.549,10 | 235,35 | 161,73 | 227,41 | 77,73 | 400,90 | 8,000 | 0,796 |
| 111Cd (KED) | 2,41 | 2,29 | 1,45 | 0,96 | 3,86 | 1,45 |  | 1,45 | 1,45 | 1,45 | 2,000 | 1,000 |
| 208Pb (KED) | 39,05 | 51,33 | 7,38 | 6,82 | 51,02 | 17,74 | 3,07 | 18,57 | 15,85 | 19,63 | 8,000 | 0,624 |
